# Orbital cellulitis unmasking disseminated TB in a kidney transplant recipient

**DOI:** 10.5588/ijtldopen.25.0315

**Published:** 2026-03-13

**Authors:** H. Ghabi, R. Belaid, S. Tlili, L. Rais, F. Attia, F. Ben Hmida, R. Abdelmalek, I. Mami, M.K. Zouaghi

**Affiliations:** 1 Nephrology Department, Rabta University Hospital, Tunis, Tunisia;; 2 Faculty of Medicine of Tunis, Tunis El Manar University, Tunis, Tunisia;; 3 Research Laboratory LR00SP01, Charles Nicolle Hospital, Tunis, Tunisia;; 4 Infectious Diseases Department, Rabta University Hospital, Tunis, Tunisia.

**Keywords:** tuberculosis, orbit, immunosuppression, solid organ transplant

Dear Editor,

We present the case of a 64-year-old Tunisian man with end-stage renal disease of unknown aetiology. He had been on haemodialysis since 2008 and underwent kidney transplantation in 2018, receiving a graft from a deceased donor. He received induction therapy with antithymocyte globulin and intravenous methylprednisolone. Maintenance immunosuppression consisted of a triple-drug regimen including tacrolimus, mycophenolate mofetil (MMF) (2 g/day), and prednisone (10 mg/day). His pre-transplant history included hypertension and a resolved hepatitis B infection. The early post-transplant course was complicated by corticosteroid-induced diabetes mellitus. In 2023, he was diagnosed with pulmonary TB. He received standard first-line anti-TB therapy, starting with a 2-month intensive phase consisting of isoniazid, rifampicin, pyrazinamide, and ethambutol, followed by a 12-month continuation phase with isoniazid and rifampicin. This regimen led to clinical improvement, negative sputum cultures by the end of the 2nd month, and radiological clearance confirmed on follow-up chest computed tomography (CT) at 1 year.

In early February 2025, he presented with fever and a 5-day history of periorbital swelling on the right side. He denied any history of cough, weight loss, nasal obstruction, or other significant clinical symptoms. Physical examination revealed purulent discharge in the right eye with an erythematous swelling of the upper eyelid, which spontaneously ruptured, releasing discharge through a fistulous tract. A less prominent swelling was also observed in the infraorbital area (Figure Panel A). Ophthalmologic evaluation revealed preserved visual acuity in both eyes. Intraocular pressures were within normal limits bilaterally. On slit-lamp examination, the right eye showed mild conjunctival hyperaemia, but there were no signs of anterior chamber inflammation. The cornea appeared clear. Fundoscopy revealed a healthy optic disc and normal retinal vasculature, with no evidence of choroiditis, retinitis, or vitritis. Importantly, there were no ocular signs suggestive of TB, such as uveitis, choroidal granulomas, serpiginous-like lesions, or Bouchut nodules. Nasal endoscopy did not reveal any abnormalities. Baseline laboratory results are detailed in Table.

**Figure.**
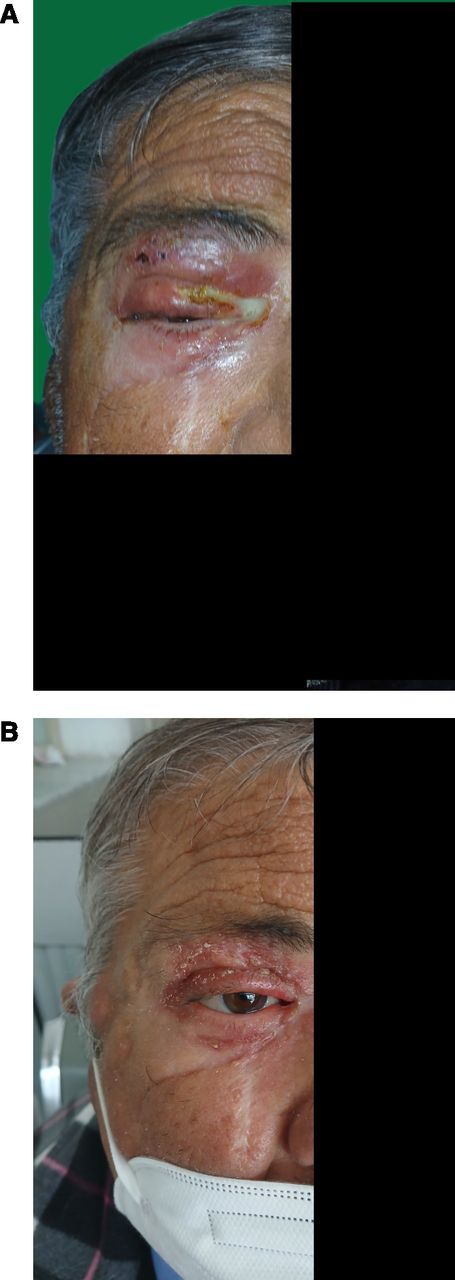
Orbital TB. **A:** Baseline appearance. **B:** Evolution following 2 months of treatment.

**Table.** Baseline laboratory results.

Parameter	Value
Serum creatinine (µmol/L)	103
Calcium (mmol/L)	2.23
C-reactive protein (mg/L)	243
Procalcitonin (µg/L)	0.02
Albumin (g/L)	35
Fasting blood glucose (mmol/L)	5.4
HbA1c (%)	9.7%
Total cholesterol (mmol/L)	2.9
High-density lipoprotein cholesterol (mmol/L)	1.6
Uric acid (mmol/L)	0.4
Haemoglobin (mg/dL)	11.2
Leucocyte count (×10^3^/µL)	5.4
Lymphocyte count (×10^3^/µL)	0.4
Tacrolimus trough level (ng/mL)	5
Blood culture	Negative
Human immunodeficiency virus serology	Negative
Hepatitis B virus DNA polymerase chain reaction	Negative
Blood polymerase chain reaction for cytomegalovirus	Negative

A contrast-enhanced CT scan of the right orbit revealed right-sided preseptal orbital cellulitis. There was no evidence of sinus pathology. Empirical broad-spectrum antimicrobial therapy with piperacillin–tazobactam and teicoplanin was initiated, but the clinical course was unfavourable. A skin biopsy of the infraorbital lesion was therefore performed. Histological analysis demonstrated a granulomatous inflammation without caseous necrosis, surrounded by epithelioid cells and multinucleated Langerhans giant cells, findings highly indicative of TB. A thoraco-abdominopelvic CT scan was performed to investigate other sites of TB involvement. It revealed right-sided cervical lymphadenopathies. Pulmonary findings included nodular and micronodular opacities in the right Fowler segment. Additionally, there was evidence of terminal ileitis associated with mesenteric lymph nodes, exhibiting features suggestive of TB. Diagnostic analysis of bronchoalveolar lavage samples confirmed the presence of *Mycobacterium tuberculosis* (MTB) by polymerase chain reaction. The diagnosis of TB was established. The drug susceptibility testing did not indicate any resistance to the standard anti-TB medications. The patient was initiated on a treatment regimen for TB, which included rifampicin, isoniazid, pyrazinamide, and ethambutol. Tacrolimus doses were increased approximately five fold to maintain trough levels between 3 and 4 ng/mL. The dose of MMF was reduced to 1 g per day, and corticosteroid therapy was maintained. After 2 months of treatment, the patient showed clinical improvement (Figure Panel B), with resolution of fever, enhancement of the ocular condition, and normalisation of C-reactive protein level. No evidence of TB within the patient’s family was identified as a potential source of infection.

TB remains a significant global health concern, particularly among immunocompromised individuals such as solid organ transplant (SOT) recipients.^1,2^ The prevalence of active TB among SOT recipients ranges from 1.2% to 6.4% in developed countries but can reach up to 12% in high-burden regions.^2^ Immunosuppression not only heightens susceptibility to TB but also modifies its clinical presentation, often resulting in atypical or non-specific symptoms that can delay diagnosis.^3^ Ocular TB is an uncommon but potentially serious manifestation of the disease.^4,5^ It can present with a diverse array of clinical features. *MTB* can affect ocular tissues through several distinct pathways. Primary ocular involvement may occur via direct inoculation of the conjunctiva or adnexal structures. More commonly, ocular manifestations arise from haematogenous dissemination of MTB originating from a pulmonary or extrapulmonary focus. Some cases of Ocular TB result from a delayed hypersensitivity reaction, where ocular inflammation is triggered by immune responses to MTB antigens.^4^ Eyelid TB occurs most frequently in the paediatric population. It may present in various forms, including lupus vulgaris, characterised by reddish-brown nodules, persistent chronic blepharitis, or diffuse swelling that clinically mimics the appearance of preseptal cellulitis.^4^

Orbital TB remains exceptionally rare, even in regions where TB is endemic, with only a few cases documented in the literature.^5–9^ To our knowledge, this is the first reported case in a kidney transplant recipient. According to our review, orbital TB may present as an isolated condition or occur alongside TB affecting other organs, including lymph nodes, skin, and lungs.^5–7,10,11^ This highlights the importance of a thorough clinical and radiological assessment, particularly in cases of orbital cellulitis with atypical features or unfavourable progression despite standard antibiotic therapy, especially in immunocompromised individuals.

In SOT recipients, both the diagnostic approach to suspected active TB and the appropriate selection of therapeutic regimens are critical considerations.^2^ In the present case, histopathological examination revealed clusters of epithelioid macrophages and granulomas lacking well-formed caseous necrosis. However, these findings remained consistent with a diagnosis of TB, as the inflammatory response may have been altered by the patient’s immunosuppressed state.^12^ The optimal duration of therapy in SOT recipients has not been clearly established, although prolonged treatment courses have been reported in the literature.^2,3,13^ In our patient, the first episode of pulmonary TB was treated for a total of 14 months, with clinical improvement and negative sputum cultures by the end of the 2nd month, raising the question of whether longer treatment could have prevented reactivation. Finally, this case highlights the need for individualised immunosuppressive strategies in SOT recipients with a history of TB to reduce recurrence risk. While the mTOR pathway has been studied in relation to MTB host response, enhancing autophagy for bacterial clearance, the role of mTOR inhibitors in preventing TB recurrence in SOT recipients remains unexplored.^14^

TB remains a significant clinical challenge in SOT recipients, particularly in regions with high endemicity. This presentation underscores both the diagnostic complexity and the therapeutic difficulties associated with TB in immunocompromised patients. A multidisciplinary approach involving infectious disease specialists and transplant physicians is crucial to ensure timely diagnosis and effective management.
